# Exploring the identity development of the budding neuroscientist at postgraduate level: a mixed-method study with perspectives from alumni and academics

**DOI:** 10.1186/s12909-022-03758-0

**Published:** 2022-10-28

**Authors:** Stefano Sandrone, Iro Ntonia

**Affiliations:** 1 Department of Brain Sciences, Faculty of Medicine, Imperial College London, London, UK; 2 Centre for Higher Education Research and Scholarship (CHERS), Imperial College London, London, UK

**Keywords:** Neuroscience, Neurology, Neuroscience education, Medical education, Identity, Identity development

## Abstract

**Introduction:**

Neuroscience represents one of the most exciting frontiers in scientific research. However, given the recency of neuroscience as a discipline, its inter- and multi-disciplinary nature, the lack of educational research on brain science training, the absence of a national or global benchmark and the numerous neuroscience subfields, the development of the academic neuroscientist identity across career stages remains obfuscated. Neuroscience is not predominantly taught at the undergraduate level but presents as a postgraduate specialism, accepting graduates from a wide range of primary disciplines.

**Methods:**

This work represents the first mixed-method study exploring the development of the neuroscientist identity at the postgraduate level at a high-ranking, research-intensive UK University. It combines responses from standardised self-efficacy and professional identity questionnaires and qualitative data from nineteen semi-structured interviews with alumni and academics.

**Results:**

Key findings on influences, identity transitions, curricular skills and sense of belonging have been discussed. The results obtained can be mapped against the theoretical framework proposed by Laudel and Gläser in 2008, although some minor changes to the model have been suggested.

**Discussion:**

Implementing active learning strategies and experiential assessments, designing mentoring opportunities and creating spaces for interaction can favour the transition from students to neuroscientists and contribute to an inclusive and diverse neuroscientific community.

## Introduction

Neuroscience represents one of the most exciting scientific frontiers. Yet, it is only since the 1960s that it has been defined as a distinct discipline [1]. The word *neuroscience* was coined by biophysicist Francis Schmitt [2] as an umbrella term under which to publish a bulletin with ‘*provocative thoughts on everything from synaptic function to artificial intelligence*’ ([2], p 598) and promote a fundraising campaign for a summer school for budding neuroscientists [2]. Since the discipline’s inception, defining neuroscience as a discipline has been strictly linked with the training needed to become a neuroscientist.

But the development of neuroscience identity is more complex than other disciplines within the science, technology, engineering, and mathematics (STEM) remit for five key reasons. First, neuroscience is a relatively young discipline. Second, it is intrinsically multi- and inter-disciplinary, as it integrates knowledge from several disciplines [3, 4]. Third, the number of fields and subfields within neuroscience makes it challenging to consider this discipline a monolithic unit. Fourth, there is a lack of national or international benchmarks for the neuroscience curriculum. Fifth, incoming students’ scientific backgrounds are incredibly diverse, including medicine, pharmacy, biochemistry, biotechnology, engineering, biology, psychology and philosophy. Few studies exist on identity, influences and sense of belonging at the postgraduate level in neuroscience, and no evidence has been collected on how master programmes contribute to the development of neuroscientists’ identity and how this identity evolves across academic career stages.

### Aims and research questions

Although neuroscience is a ‘*singular label (…) it embraces a plurality of disciplines*’, and ‘*molecular and cognitive neuroscientists scarcely speak a common language*’ ([2], p 599). This study explores neuroscience identity by assessing self-efficacy and professional identity among alumni and academics in a high-ranking research-intensive University of the United Kingdom (UK) via a mixed-method approach. These are some of the research questions: how does the neuroscientist identity develop at the postgraduate level? What influences the students’ transition to becoming a neuroscientist, and how does this happen? How do self-efficacy and professional identity contribute to it? Which aspects of the students’ budding scientific identity help them perceive their strengths and determine their identity? What are the skills that students and academics value the most? Which skills should be developed to mentor stronger generations of neuroscientists? Do academics feel they belong to the neuroscience community? What are its boundaries, and how do students and academics perceive these?

## Methods

This study used mixed methods combining two validated surveys with nineteen semi-structured interviews. This study employed an explanatory sequential design, whereby participants for the qualitative component were directly recruited from the pool of the scale completers. This project adhered to British Educational Research Association (BERA) guidelines [5] and received ethical approval from the Education Ethics Review Process at Imperial College London (EERP2021-012). All methods were carried out in accordance with relevant guidelines and regulations. Informed consent was obtained from all subjects.

### Participants

Eight alumni (six females and two males) and eleven academics (all males) from a UK-based STEM-intensive institution participated in the study. *Alumni* refers to former students who have completed the Master of Science (MSc) in neuroscience at a STEM-intense institution within the last four years. Furthermore, we split the academics into two different groups, according to their seniority within the neuroscience field as indicated by the academic ranking. In the UK system, Lecturer (equivalent to Assistant Professor in the USA) and Senior Lecturer (Associate Professor) identify the first two stages of the academic ranking after PhD and postdoc, whereas the ranks of Reader and Full Professor are the subsequent stages of the academic ranking. Therefore, in this manuscript Lecturers and Senior Lecturers have been grouped as junior academics, whereas Readers and Professors have been grouped as senior academics.

### Surveys

Participants completed two validated surveys (20-item long combined), requiring less than five minutes to complete online via Qualtrics: the *New General Self-Efficacy Scale* [6] and the modified version of the *Professional Identity Scale* (as used in the New Generation Project Longitudinal Study by Rebecca Foster and colleagues). The order of the answers in the second scale (*strongly agree* to *strongly disagree*) was the opposite of the first (*strongly disagree* to *strongly agree*). This format was implemented to ensure that participants maintained attention.

The questions of the *New General Self-Efficacy Scale* [6] were:

1. I will be able to achieve most of the goals that I set for myself.
2. When facing difficult tasks, I am certain that I will accomplish them.
3. In general, I think that I can obtain outcomes that are important to me.
4. I believe I can succeed at most any endeavor to which I set my mind.
5. I will be able to successfully overcome many challenges.
6. I am confident that I can perform effectively on many different tasks.
7. Compared to other people, I can do most tasks very well.
8. Even when things are tough, I can perform quite well.

Response format included: strongly disagree, disagree, neither agree nor disagree, agree, strongly agree. This scale uses a 5-point Likert-type rating scale (1 = strongly disagree; 2 = disagree; 3 = neither agree nor disagree; 4 = agree; 5 = strongly agree) and a higher score means higher self-efficacy. An average score was calculated for each group of participants for each question to allow a granular comparison.

The questions of the *Professional Identity Scale* were:

1. I feel like I am a member of this profession.
2. I feel I have strong ties with members of this profession.
3. I am often ashamed to admit that I am studying for this profession.
4. I think of myself as a typical example of an average member of this profession.
5. I find myself making excuses for belonging to this profession.
6. I try to hide that I am studying to be part of this profession.
7. I am pleased to belong to this profession.
8. I am a person who criticises the profession for which I am studying.
9. I can identify positively with members of this profession.
10. When I hear someone who is not a member of this profession criticising this profession, I feel personally criticised.
11. Being a member of this profession is important to me.
12. I feel I share characteristics with other members of this profession.

Response format included: strongly agree, agree, neither agree or disagree, disagree, strongly disagree. Similarly to the previous scale, also this one was a 5-point Likert-type rating scale. However, differently from the previous one, this scale had a mix of direct and indirect (numbers 3, 5, 6, 8) answers.

### Semi-structured interviews

Interviews took place via Microsoft Teams between November and December 2020, with individual interviews lasting no longer than sixty minutes.

Indicative questions for the alumni:

1. Who would you say has influenced your career development and the way you approach the discipline and why?
2. How did this person/these people contribute to your growth as a neuroscientist?
3. What did you value the most from your MSc and why?
4. How did attending the MSc affect you?
5. To what extent did you feel integrated with the MSc cohort?
6. How do you feel about being an alumna/alumnus of the programme now?
7. What are the skills you think a neuroscientist should have?
8. Which changes would you suggest to the MSc curriculum?

Indicative questions for the academics:

1. Who would you say has influenced your career development and the way you approach the discipline and why?
2. How did this person/these people contribute to your growth as a neuroscientist and why?
3. Tell me a little bit about your postgraduate study experience: which elements of it did you value the most? Has this changed at all over time?
4. How (if at all) would you say your postgraduate studies contributed to your identity as a neuroscientist?
5. What are the skills you think a neuroscientist should have?
6. How do you feel about being a member of the neuroscience community now?
7. Which changes would you suggest to the MSc curriculum?

Follow-up questions on aspects that did not seem clear in the first instance or that could have been further explored for educational interest and relevance to the research project [7, 8] were asked by the interviewer.

### Thematic analysis

Audio recordings were anonymised and sent to a third-party transcription service (Way With Words Ltd.) for verbatim transcription. For the analysis of the interviews, the structured thematic analysis approach [9] was followed, whereby the emergence of themes was guided by the questions, which acted as filters [8]. Immersion and familiarisation with the data were needed before generating codes (of any size, from words to sentences) using the open and axial coding process [10], which allowed the identification of first and second-order codes. While open coding permitted the deconstruction of the dataset, and meaningful sentences/relevant themes (phenomena) were highlighted with colours, axial coding allowed the re-construction of the main topics in new, meaningful ways.

## Results

### Self-efficacy and professional identity

**Fig. 1:**
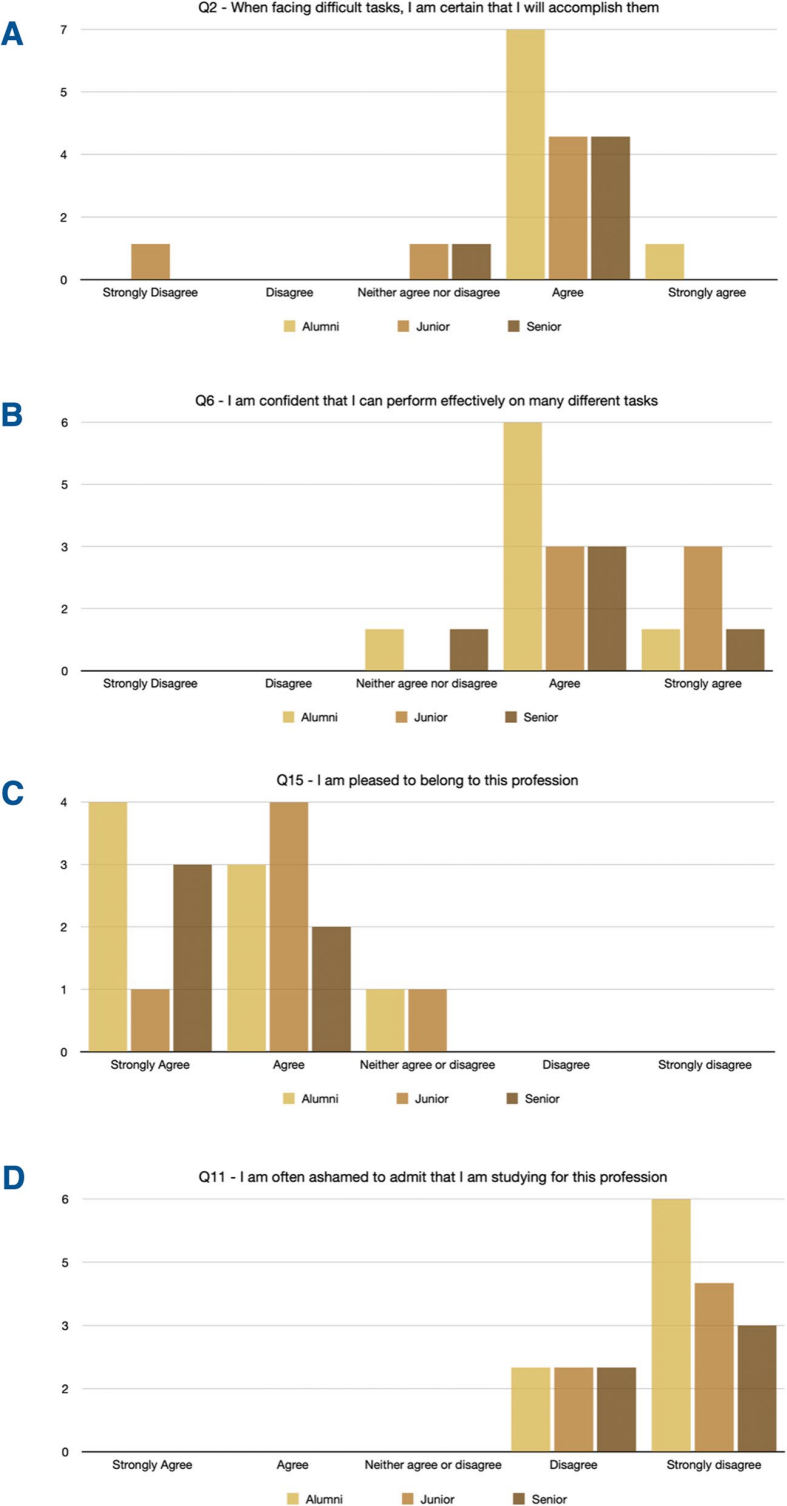
Participants’ answers to questions from the *New General Self-Efficacy Scale* (Q2 and Q6) and the *Professional Identity Scale* (Q15 and Q11)

Senior academics scored the highest self-efficacy (total average for alumni vs junior academics vs senior academics: 31.5 vs 30.5 vs 32.2). The alumni were more confident than junior and senior academics in accomplishing difficult tasks, and junior academics were less confident than senior academics (average: 4.125 vs 3.33 vs 3.8; ‘*Question 2—When facing difficult tasks, I am certain that I will accomplish them*’, Fig. 1A). However, junior academics were slightly more confident than alumni and senior academics in performing *effectively* on different tasks (average: 4 vs 4.5 vs 4; ‘*Q6—I am confident that I can perform effectively on many different tasks*’, Fig. 1B).

**Fig. 2:**
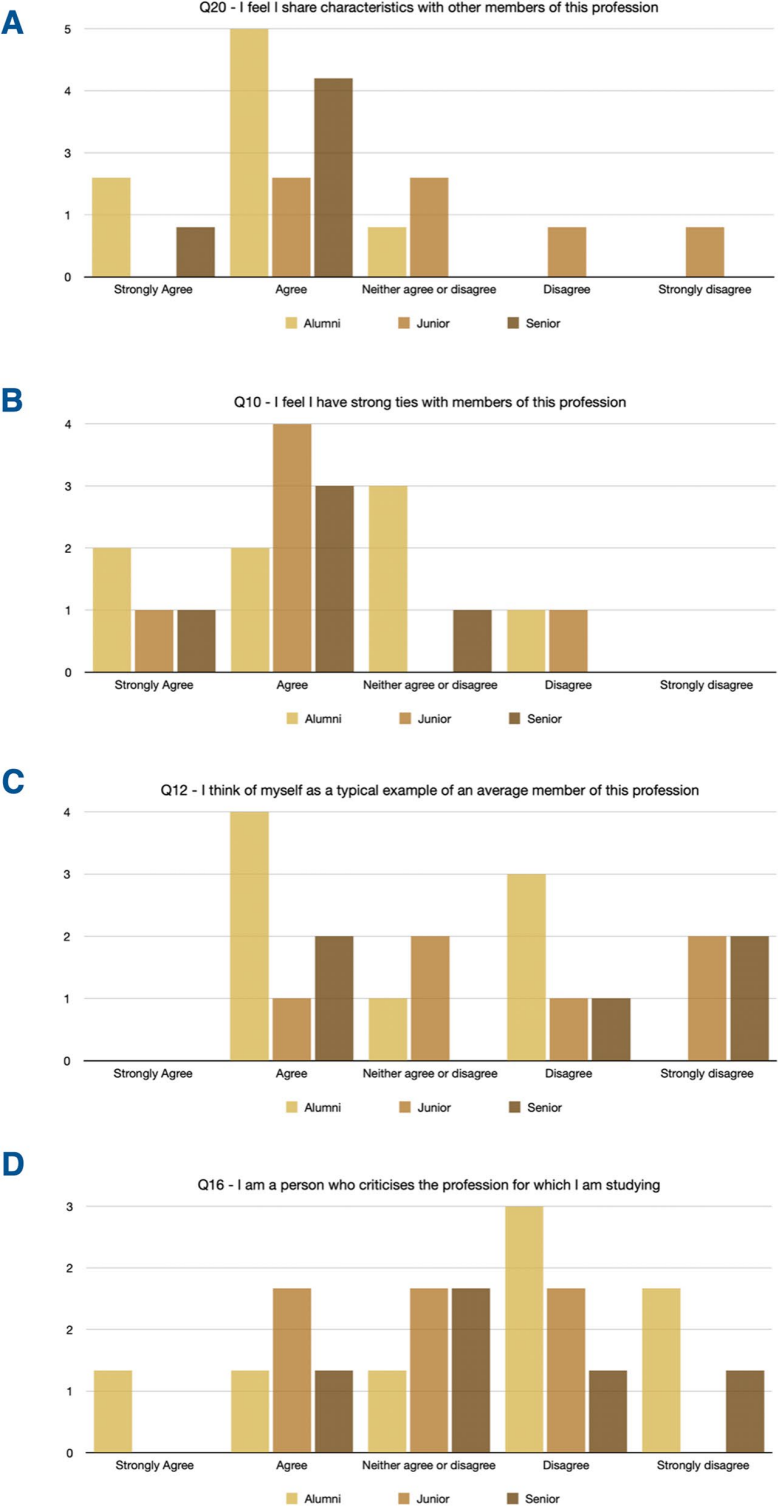
Participants’ answers to questions from the *Professional Identity Scale*

The overall score for the Professional identity Scale was higher for the alumni than the academics (total average for alumni vs junior academics vs senior academics: 47.875 vs 43.494 vs 46.9)’. While most participants were pleased to belong to the profession (‘*Q15—I am pleased to belong to this profession*’, Fig. 1C) and were not ashamed of studying for their profession (‘*Q11—I am often ashamed to admit that I am studying for this profession*’, Fig. 1D), junior academics displayed a weaker sense of belonging than senior academics. Moreover, there was a stronger sense of *shared characteristics* in the alumni than in junior academics (‘*Q20—I feel I share characteristics with other members of this profession*’, Fig. 2A). Alumni’s ties with members of the profession were perceived as existing, but not that strongly (‘*Q10—I feel I have strong ties with members of this profession’*, Fig. 2B). Mixed feelings about being a ‘*typical example of an average member*’ of the profession (‘*Q12—I think of myself as a typical example of an average member of this profession*’, Fig. 2C) and criticising the profession (‘*Q16—I am a person who criticises the profession for which I am studying’*, Fig. 2D).

### Thematic analysis

**Fig. 3:**
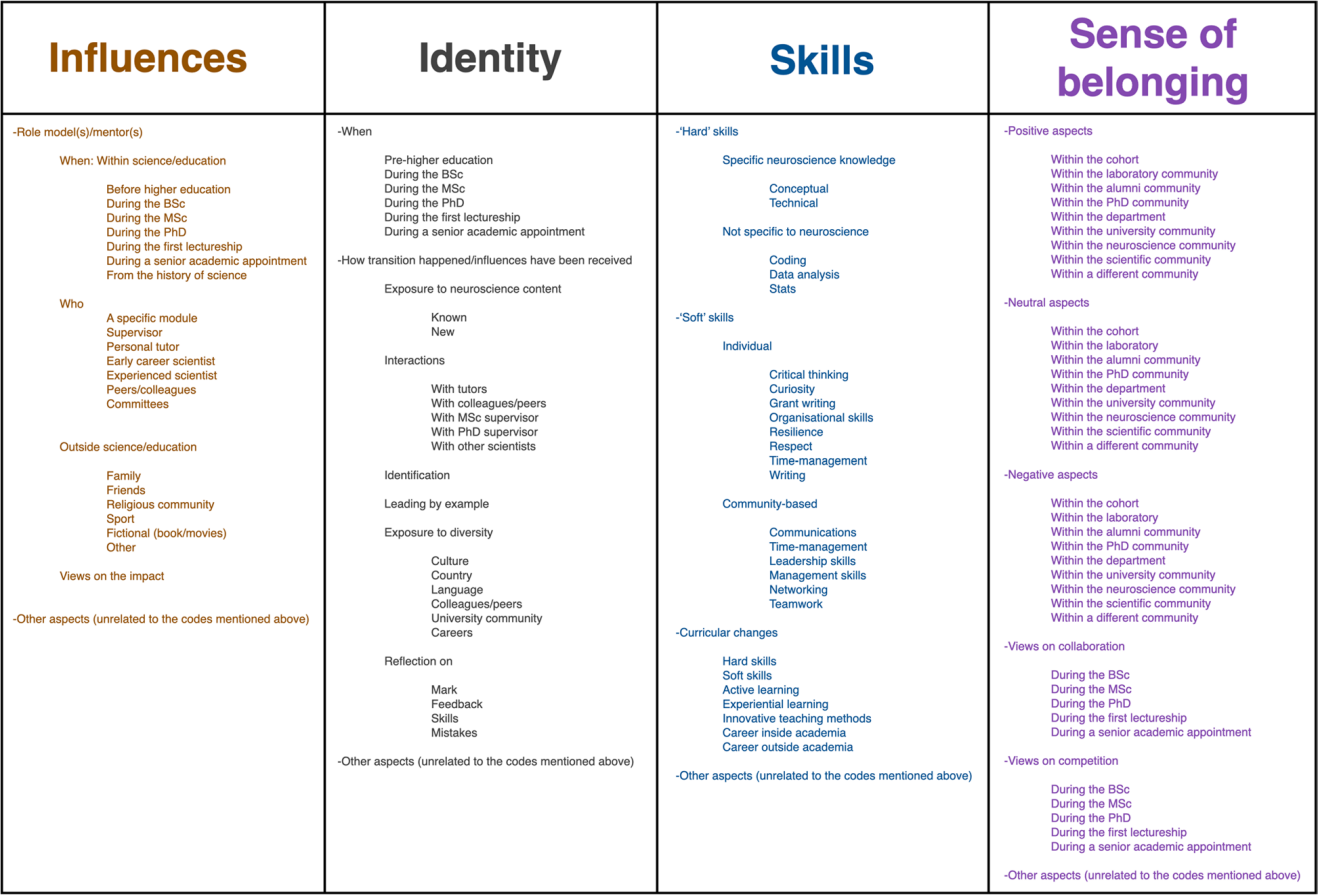
Four themes emerged from the thematic analysis: Influences, Identity, Skills and Sense of belonging

Four themes emerged: Influences, Identity, Skills and Sense of belonging (Fig. 3).

### Influences: who, when, what

#### Alumni

Most of the alumni mentioned their supervisors, tutors, teachers and educators as critical influences in their career development and approach to the discipline; a minority also mentioned the family environment, the support received and the presence of healthcare professionals in the family. Several alumni spontaneously used the terms *role models* or *mentors* and said the act of observing colleagues at work was inspirational per se. However, they wished to have more contact with them: ‘*You see what they do, how they approach the subject and their duties*’ and ‘*what a neuroscientist is about*’ (A1). A2 highlighted the dedication of fellow students and teaching staff, and mentioned the exposure and determination as ‘*very inspiring to see*’. A4 said that a positive daily influence made the whole endeavour more approachable. A7 reiterated the importance of ‘*being challenged*’ and said that ‘*it’s easier to have a role-model that’s closer to your stage*’ because ‘*Principal Investigators seem so far away*’ and unapproachable.

#### Junior academics

Only two junior academics out of six cited peers as notable influences. Junior academics were almost all concordant in indicating that PhD and postdoc supervisors/co-supervisors were key influences. Mentors created ‘*opportunities*’ and favoured networking (J1). In addition to the love for the subject and the inspirational aspects already seen with the alumni, J1 learnt elements of compassion and empathy (essential for a clinician-scientist). J2 indicated the postdoc as the key moment, when ‘*you decide what kind of scientist you want to be and what kind of thinking you will have*’. Two junior academics said the influence of mentors and role models has been less impactful than what could have been imagined at the beginning of the career. In J6’s words: ‘*I've learnt just from the mistakes that I've made myself.* (…) *if you're going to go down in flame, go down in your own plane’.* Even more explicitly, J3 stated: ‘*I don’t feel like I have had a specific role model. I tried along my journey to learn from everyone. (…) it felt like it was never a done job, the path that I have followed. But in every step, I tried to almost, as we say, (…) steal from people things that I like’.*

#### Senior academics

PhD and postdoc supervisors were named as influences leading by example (S4: ‘*I see that individual as an exemplar in everything that they’ve done and their approach to neuroscience’*) by all participants but one (S1). Compared to their junior colleagues, senior academics put more emphasis on non-research-related skills. The rigour of research, leadership skills and vision to get things done were listed by S1. S2 added the ability to think ‘*outside the box*’, ‘*sense of adventure*’, ‘*relating to the others, to the team*’, ‘*sense of self-confidence*’. Mentors and role models were perceived as ‘*enablers of what was inside (…) me*’ (S2). For S3, ‘*open, critical mind and willingness to listen to everyone, and to consider very openly every deviation from the normal science path were very enlightening to me’*. For S4, it was the ability to look at the bigger picture. Only one senior academic (S2) cited colleagues who led by example: ‘*I always thought, and I still think of them, as good examples that I would like to imitate and, potentially, even going to improve upon*’. Only one senior academic cited the students as a positive influence (S3, ‘*for their successes, for their smart moments, as well as their failures, as well as my own*’).

### Identity and postgraduate studies: the ‘how’

#### Alumni

The sense of identity resulted from a combination of factors:

i) confirmation of interest in neuroscience and easiness in the ‘*transition*’ (A3), coupled with the ‘*directionality*’ received (A5). These elements ‘*solidify the intention of becoming neuroscientist, representing a very important step closer towards that title*’ (A6).
ii) The ownership and the confidence felt at the research project stage.
iii) Personal development deriving from leaving the comfort zone, living in a different country and studying in a foreign language (A7 and A4).

Overall, the course seemed to have shaped their identities, and ‘*left a very, very visible mark on me, which I like. And it has also structured my mindset (…), my attitude, it has really framed it. So, it’s not just about how I feel about it, but also what I reflect, I think, of my experience*’ (A1).

#### Academics

Two junior academics considered postgraduate training (PhD and postdoc) essential in teaching them skills and managing emotions: ‘*my identity was created essentially by the research and the papers that I developed during my postdoc*’ (J5); ‘*by giving this toolbox of skills, by trying to instil a sense of not getting flustered*’ (J6). J2 highlighted non-scientific factors, such as the places and the cultures encountered. For two academics, the personal identity was already more prevalent than the one given by the postgraduate training, as they already knew what they wanted to study. Two senior academics out of five identified the PhD, not the full postgraduate training, as the turning point identity-wise for reasons linked to the learning of technical skills and the chance to jump on the academic ladder. ‘*Without the PhD I would not have gotten into academia, so that was absolutely the fundamental thing. Learning those techniques in the PhD (…) jumpstarted the whole career’* (S1). S5 defined the PhD as ‘*the passport to independent science* (…) *it’s almost irrelevant what the topic is, it’s the way you’ve approached it, the way you’ve thought it through and the way you’ve presented it*’. S3 claimed the entire postgraduate training mattered for approximately 80%, whereas for S4, the postgraduate training mattered only ‘*moderately*’.

### Skills from the MSc to the world

**Fig. 4:**
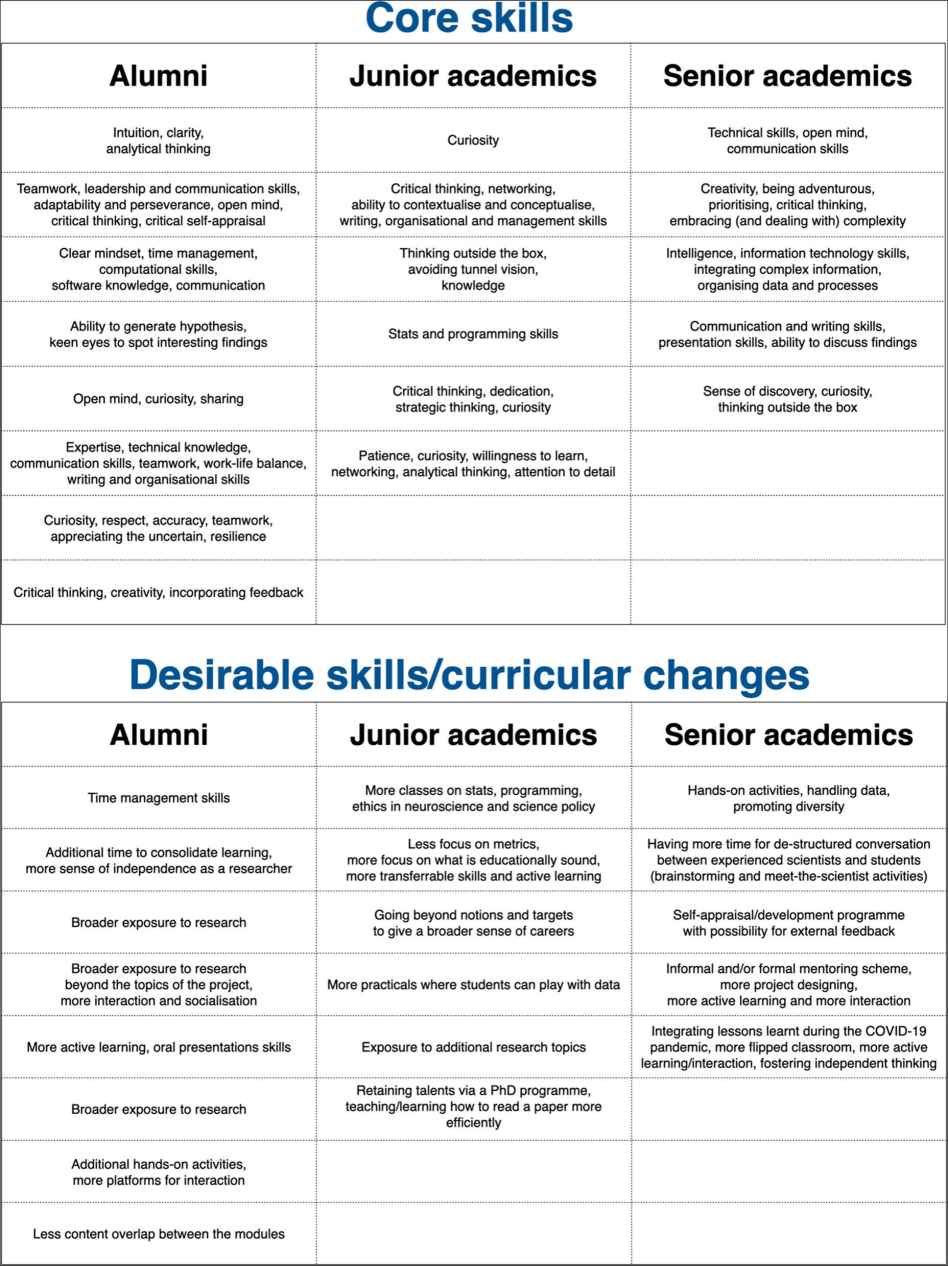
Top: core skills identified by alumni and academics. Bottom: desirable skills/curricular changes identified by alumni and academics. Each box summarises key concepts voiced by a participant

There were substantial overlaps among the core skills identified by alumni and academics (Fig. 4, top), converging on the importance of neuroscience knowledge, critical thinking, curiosity/open mind, teamwork and transferrable skills. However, the junior, but not the senior, academics highlighted two further sets of skills as essential: networking and management/leadership skills. In contrast, the alumni lacked a broader sense of community outside the laboratory. Junior academics seemed more aware of neuroscience's social/community dimension and the nature of social/scientific skills. When asked about how the training for the next generations can be improved, the social/interacting element emerged (Fig. 4, bottom).

### Desirable skills/curricular changes

A4 asked for more events and space to interact with scientists ‘by grabbing a coffee’ or ‘chatting for ten minutes’. A5 underlined the importance of ‘*actively doing and delving into something*’. Albeit with less emphasis, A3, A4 and A6, argued for additional neuroscience topics. Junior academics advocated the integration of more hands-on activities. J4 was very vocal: ‘*Get people playing with data*’. J1 encouraged the introduction of more classes on stats, programming, ethical aspects of neuroscience and science policy. A broader career-related perspective that goes beyond marks to favour interactive teaching methods such as active learning was emphasised by J2. Even senior academics were in favour of experiential, active and interactive learning. S5 championed a more widespread adoption of the flipped classroom and active learning because students ‘*come along much more engaged and ready to ask questions if you’ve made them start thinking about the topic beforehand’.* S2 suggested having ‘*de-structured conversation between an experienced scientist, PI and students (…) sharing the broader vision and the broader dreams and challenges that students will face later in life (…) having some kind of meet-the-scientist, (…). Talking about (…) opportunities in your career (…) What are the critical hurdles? How do you fulfil your dreams?*’. S4 mentioned the advantages of inserting a formal or informal mentoring scheme.

### Sense of belonging

#### Alumni

At least to some extent, the alumni bonded within the cohort. A1 mentioned a *fil rouge* within the cohort, despite the different scientific background. Many alumni appreciated group works during the first months of the course as a primer for the collaborative nature of science as they ‘*made you feel very connected*’ (A7). While sometimes it was difficult to work optimally with every colleague, A2 emphasised how formative it could be to know that ‘*you’re not the only one in this boat and you’ve got other students with you that are going through the same process*’. Other bonding factors were meeting colleagues, even briefly, before the lecture, or socially at events or in the common space on the campus.

#### Junior academics

In response to the question ‘*How do you feel about being a member of the neuroscience community now?*’, there were three enthusiastic answers among the six junior academics: neuroscience community seems to be a good place for networking. The main reasons for not being that happy were the low gratification and the limited social recognition attached to the scientist status. As explained by J2: ‘*low reward rate (…) Academics and scientists (…) are paid less and there are challenges (…) the level of anxiety among scientists is more than any other field’*. J5 thinks the neuroscience community is ‘*an amorphic terminology*’, and J4 voiced a ‘*strong affinity*’ with neuroscientists working on the same topics, but not with those who are experimentally far away.

#### Senior academics

Two academics expressed positive feelings about being part of the neuroscientific community. In contrast, the other four emphasised the role of small communities within the larger neuroscience community, highlighting a different facet of this phenomenon. S2 related to the act of caring about peers, because the sense of belonging ‘*gives you a sense of fulfilment (…) I feel good about it, but I’m not particularly proud of the fact that I’m a neuroscientist. You feel you belong because of your achievements. You don’t belong because of your title or (…) your position*’. S3 used an astronomic metaphor: ‘*it’s a bit more difficult to relate strongly to every member and every (…) subdivision (…) of such a big field. If we break it down (…) we [see] each of them is a constellation with many stars. There are some stars in neuroscience that I have nothing to do with. Their light doesn’t reach me. I’ve never turned that way. Put it whatever way you like, but these stars are alien worlds to me’*.

## Discussion

From the quantitative analysis, senior academics scored the highest self-efficacy, whereas alumni reported the highest score on the Professional Identity Scale. Although most participants were pleased to belong to the profession, there was a stronger sense of *shared characteristics* in the alumni than in junior academics, who had a weaker sense of belonging than senior academics. From the qualitative part of the study, it emerged that the identity of each group had been shaped by mentors they met during their learning journey. Alumni and junior academics linked their own identity to personal development and pragmatical skills learnt, valued networking and voiced the importance of learning by doing. Academics recognised PhD and/or postdoc training as a defining moment in their identity development. However, senior academics put less emphasis on networking but recognised the importance of non-research-related skills (i.e., leadership skills) and having the ability to look at the bigger picture.

### Identity development at the alumni’s stage

Alumni’s higher self-efficacy compared to junior academics was surprising, but this might be linked to the skills-related and personal growth during the master's. For several alumni, the MSc experience made them more resilient and proactive. This aligns well with reports showing that self-efficacy and resilience are linked: self-efficacy (and self-care) partially mediates the relationship between attachment security and resilience [11]. Their professional identity seems to be more defined than junior academics: alumni scored a higher sense of sharing characteristics than junior academics, despite having fewer ties with colleagues, given their early career stage: their identity has been consolidated by the MSc. Still, it has not yet been put into an existential crisis by the difficulties of the PhD. Only a few started their doctorate at the interview, and the PhD represents a crisis step for many young scientists. It has been defined as the PhD identity crisis, both experimentally [12] and writing-wise [13], by which the students ‘*come to know their subject and their scholarly selves*’ ([14], p 863).

### Identity development at the academics’ stage

As ‘*higher education is a turbulent sector*’ ([15], p 998), the initial transitions into the academic appointments of junior academics represent a stage with ‘*considerable consequences for career development and willingness to remain within*’ the profession ([15], p 998). Junior academics reported lower self-efficacy compared to alumni. On the one side, they still have to grow to reach the top level and are aware of the difficulties of starting a laboratory. Concerns, including the limited availability of tenure jobs [16] and the difficulty of securing funding for research [17] have been reported, and the situation might be further exasperated by the COVID-19 pandemic [18, 19]. On the other side, they are more confident than alumni and senior academics in performing *effectively* on different tasks. They displayed a weaker sense of belonging but, as analysed in the semi-structured interviews, they value the scientific community and feel they belong. They are willing to explore the benefits of interactions within the neuroscientific community (both in large and small niches), from networking to establishing new collaborations. They are keen on looking for new opportunities, networking and collaborations even in sub-fields away from their day-to-day research topics. In their reflections, the term *collaboration* appeared more often than *competition*. This aligns well with Holley (2018): ‘*Throughout this transition, early-career scholars ideally find a place within their respective scientific communities and strengthen the foundation for their career*’ ([20], p 109).

In contrast to junior academics, senior academics’ professional identity seems more defined: they are aware of their progress and feel recognised. It is impossible to establish if the higher self-efficacy (compared to junior academics) was *the* driving force pushing them to the top or if they have high self-efficacy because of their appointment. Whatever the cause, upon reaching the top academic rank, they tend to follow research updates from their neuroscience niche, where they are leaders. Despite being culturally interested in the latest neuroscientific findings, they do not feel they belong to neuroscientific communities away from their *tribe*.

### Hands-on activities to nurture becoming and belonging

Working in teams during the master’s made the alumni feel part of a real, authentic community. They recognised the importance of teamwork as a proxy of what working in the scientific community might look like (with pros and cons), despite not liking all the working styles they came across. This aligns well with studies describing how students and early career researchers in STEM appreciate being exposed to collaborative research practices and for the ‘*application of knowledge in real-world, complex situations*’ ([20], p 107; [21, 22]). In a community of practice, ‘*novices and experts work together (…) to learn and connect STEM content and skills*’ ([23], p 2). However, this exposure should be sustained over time: limited ‘*research-intensive phase prior to normal academic employment*’ ([24], p 387) and lack of time for research may hinder the transition.

Alumni and academics highlighted the importance of learning by doing. Active learning reportedly increases students’ performance in STEM [25], showed benefits across educational levels [26] and in many disciplines, including neurology [27] and psychiatry [28]. It narrows achievement gaps for underrepresented students and can promote equity in higher education [29, 30], maximises their learning online [31], increases students' satisfaction with individual and group activities [32]. Active learning promotes *belonging* and *becoming* [33], which are at the core of students’ retention measures [34, 35].

### Mentoring and role models

Despite not using warm keywords in the interviews, the participants mentioned *role model* and *mentor*. Although the two words define different figures, *mentor* often recurred in the interviews. Mentorship is positively associated with the scientific successes of mentees in STEM [36, 37] and with ‘*favorable behavioral, attitudinal, health-related, relational, motivational, and career outcomes*’, each with a small effect size ([38], p 254). The key factors behind successful mentoring relationships are under-researched in neuroscience. One of the most influential papers was published only in 2018; still, the analysis of the database, which featured almost nineteen thousand researchers in STEM (and an emphasis on neuroscience), focused on the postdoc, as it was found that ‘*postdoctoral mentors were more instrumental to trainees' success compared to graduate mentors*’ ([37], p 4840). They were primarily instrumental thanks to ‘*intellectual synthesis between their graduate and postdoctoral mentors*’ ([37], p 4840) and that mentees and mentors had slightly different expertise [37].

### Professional identity development in other professions

As explained in the previous paragraphs, neuroscience has a distinct professional identity. However, there might be lessons to learn while comparing identity development across professions. For example, while analysing the experiences contributing to the professional identity development of medical school students, Kay et al., 2018 identified ‘*clinical experiences in the preclinical years*’, ‘*exposure to the business of medicine*’ and ‘*to physicians in clinical practice*’ [39]. In many ways, this overlaps with the request to be further exposed to research works via projects and interaction with principal investigators voiced by neuroscience alumni and junior academics in this study. Another piece of research surveying third- and fourth-year students at a different US medical school demonstrated that experiential learning and interaction with colleagues, patients, mentors, and role models were the key factors in shaping their own professional identity development [40]. Reflecting on these experiences can play an important role in medicine [41] and health professions too, and this can be achieved by asking students ‘*to consider how their perceptions of a profession are shaped over time*’ and ‘*how interacting with current practitioners and mentors shapes their values, beliefs, and expectations*’ ([42], p 23), although the concept of identity in the health profession is more complex as there is a ‘*continuously redefining*’ process of ‘*what professional identity looks like*’ ([42], p 12). Academics across the health professions might benefit from reflective activities too in order to achieve ‘*professional learning and academic identity*’ ([43], p 693); moreover, recently appointed lecturers in nursing, midwifery and the allied health professions felt that ‘*sustained support specifically for developing scholarship and research*’ was needed to achieve and reinforce their own identity as academics ([44], p 69).

### Stage-specific vs transversal skills for neuroscientists

Besides the core skills identified by the three groups interviewed, characterised by a substantial overlap, minor differences were found:

- alumni focused more on personal development and pragmatic skills.
- Junior academics recognised the importance of seizing every chance arising from interactions within their communities.
- Senior academics did not mention networking but valued the importance of the ‘*big picture*’, scientifically and career-wise; they offered a grand angular vision, mostly on leadership-related abilities.

**Fig. 5:**
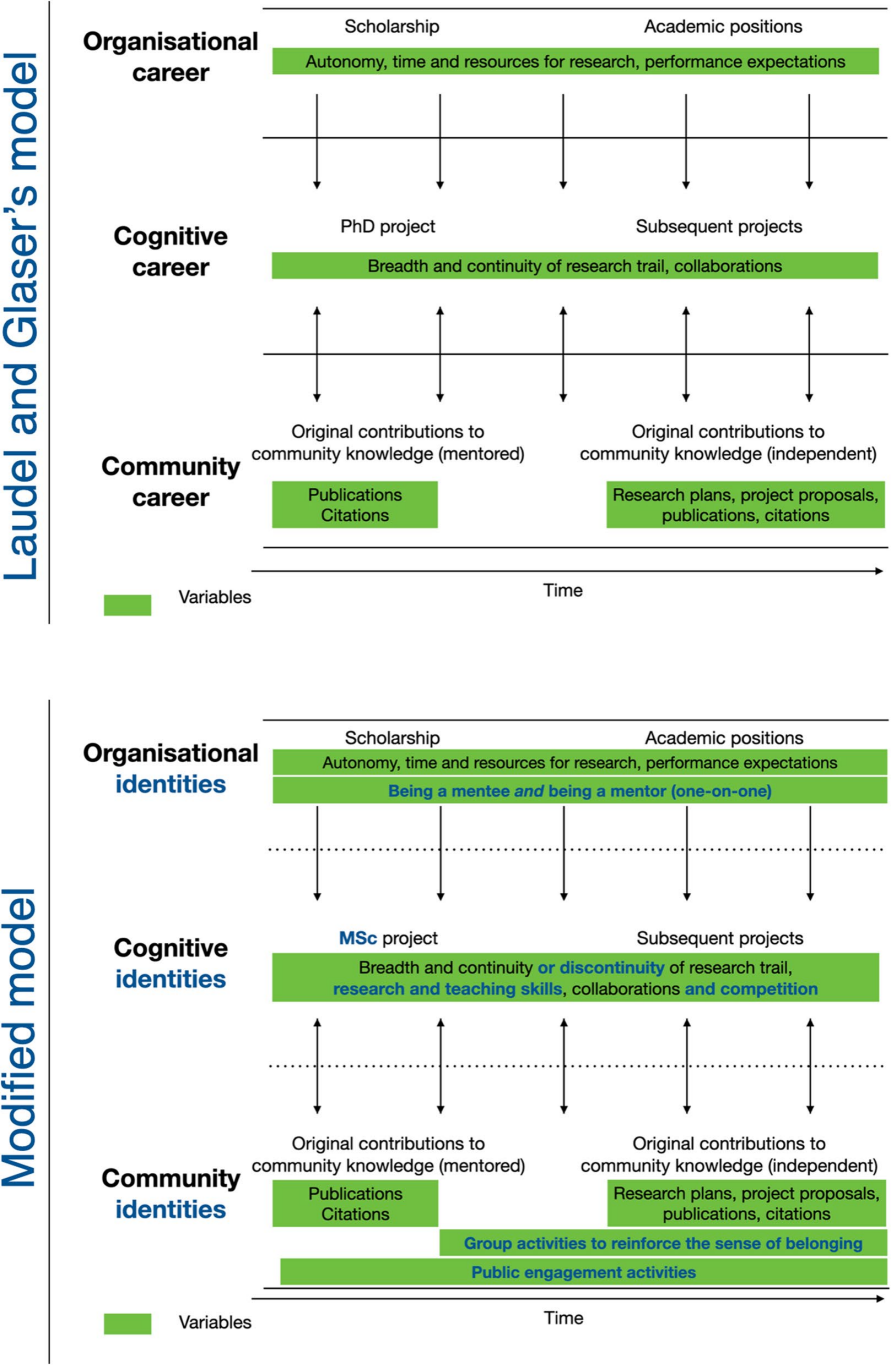
Top: Laudel and Glaser’s model of a theoretical framework of early-career researcher development [24]. Bottom: Modified version of Laudel and Glaser’s model

This again aligns well with Laudel and Glaser’s theoretical framework model of early-career researcher development [24] (Fig. 5 top), which epitomises the passage from apprentice to being colleagues, from being a supervised researcher to an independent one. It is based on three components: *cognitive career*, *community career* and *organisational career*. Interestingly, each component might be more relevant to a specific career stage:

- cognitive career for the alumni;
- community career for the junior academics;
- organisational career for senior academics.

However, some theoretical changes, to be confirmed experimentally, can be recommended (Fig. 5 bottom). At the organisational level, there might be more emphasis on *being a mentor* and *being a mentee* since the first academic steps. At the cognitive level, the framework can include master projects instead of starting at PhD stage. In parallel with the *continuity* of the research trail, *discontinuity* should be considered, within and outside the academia, as voiced by junior academics. The skills gained should be counted as factors per se. Moreover, the variable *competition* can provide a more accurate depiction of the neuroscience community. At the community level, two variables can be added: *group activities to reinforce the sense of belonging*, as a bridge connecting students and staff, and *public engagement activities* to showcase STEM diversity and create a sense of identity outside the academic community.

### Strengths and limitations

This study is the first one exploring the neuroscientists’ identity development across career levels and the first one combinedly assessing identity, influences and sense of belonging at the master level in neuroscience. The semi-structured nature of the interviews allowed a mixture of rigour in following a plot and freedom to explore the emerging topics. The use of two validated scales, the relatively high number of participants and the recruitment of participants from three different career-level groups (alumni, junior and senior academics) are strengths of this study. The strategic choice of using the General Self-Efficacy Scale instead of the Academic Self-Efficacy Scale was instrumental in gathering information not strictly related to the academic field. This study has one main limitation: all the academics recruited were male. The issue with one-gender participants is that several factors may have impacted female academics' identity development, which future studies should specifically investigate.

### Future studies

Future works should interview female academics at different career levels. Women are underrepresented in STEM [45–47], including neuroscience [48], from leadership roles [49] to citations in journals [50] and scientific awards [51]. They suffer from gender discrimination, which impacts career progression [52] and experience difficulties related to work-family balance [53]. Interviewing female academics can favour the design of schemes to overcome career challenges they face, especially considering they have been impacted more than males by the COVID-19 pandemic [19]. This could lead to analysing whether gender-related differences in academic self-efficacy in neuroscience, shown by a large meta-analysis with almost 250 studies and more than 68 thousand participants in STEM and social sciences [54] a decade ago, still exist. As the largest effect size was located for respondents of 23-year-old or more [54], whether this bottleneck exists in neuroscience (and how to neutralise it) remains to be investigated. Other works can measure self-efficacy over time and link it to academic outcomes, as in [55], and analyse whether differences exist between STEM-only and non-STEM-only institutions, and between research-intense and teaching-intense higher education providers.

## Data Availability

Data and materials are available from Dr Stefano Sandrone, the corresponding author, upon reasonable requests.
